# Association of Gastrointestinal System With Severity and Mortality of COVID-19: A Systematic Review and Meta-Analysis

**DOI:** 10.7759/cureus.13317

**Published:** 2021-02-12

**Authors:** Trishala Menon, Rohit Sharma, Geethika Earthineni, Hanan Iftikhar, Manush Sondhi, Saad Shams, Noman Khurshid Ahmed, Hira Khan, Sawai Singh Rathore, Romil Singh

**Affiliations:** 1 Family Medicine, Wheeling Hospital, Wheeling, USA; 2 Internal Medicine, Hamad Medical Corporation, Doha, QAT; 3 Internal Medicine, Sri Devaraj Urs Medical College, Kolar, IND; 4 Internal Medicine, Foundation University Medical College, Islamabad, PAK; 5 Internal Medicine, Kasturba Medical College, Manipal, IND; 6 Internal Medicine, Ross University School of Medicine, Florida, USA; 7 Medicine, Dow Medical College, Dow University of Health Sciences, Karachi, PAK; 8 Internal Medicine, Islamic International Medical College, Rawalpindi, PAK; 9 Internal Medicine, Dr Sampurnanand Medical College, Jodhpur, IND; 10 Critical Care, Mayo Clinic, Rochester, USA

**Keywords:** sars-cov-2, gastrointestinal manifestations, hepatic manifestations, covid-19

## Abstract

At present, the novel coronavirus disease (COVID-19) is causing a major pandemic. COVID-19 is caused by the Severe Acute Respiratory Syndrome Coronavirus 2 (SARS-CoV-2). In COVID-19, the patient usually presents with fever, dry cough, and respiratory manifestations. However, the involvement of other systems has also been reported in the literature. Abdominal pain, diarrhea, vomiting, and nausea are the predominant gastrointestinal (GI) manifestations underlined in the literature. We conducted a literature search using four databases (PubMed, Web of Science, Google Scholar, and Clinicaltrials.gov). Our search strategy included Medical Subject Headings (MeSH) terms and keywords for COVID-19, SARS-CoV-2, and GI system from inception to October 2020. After excluding duplicates, review articles, and non-relevant articles, we included 20 studies out of 842 articles reporting GI manifestations in COVID-19 patients. Using Cochrane RevMan version 5.4 (Cochrane, London, UK), a compute pooled analysis using a random-effect model was performed. Our study included 6,022 patients with a median age of 49.5 years. Pooled analysis via random effect model revealed an increased risk of severe COVID-19 in patients manifesting GI symptoms with an odds ratio (OR) of 2.07 (95% Confidence Interval [CI]: 1.34-3.18) with I^2^=41%). Odds of mortality in COVID-19 with GI manifestation and hepatic abnormalities included 0.92 (95% CI: 0.50-1.69) (I^2^=57%) and 1.26 (95% CI: 0.67-2.37) (I^2^=0%), respectively. Severe COVID-19 may have a strong association with GI manifestations and have a significant impact on GI practice. Holistic knowledge of the spectrum of the GI consequences in COVID-19 is crucial to get a hold of virus spread. In this article, we have summarized the association of GI manifestations in severe COVID-19 patients.

## Introduction

The coronavirus disease 2019 (COVID-19) is caused by the SARS-CoV-2 (Severe Acute Respiratory Syndrome Coronavirus 2), which originated in China in late December 2019. The World Health Organization (WHO) declared a COVID-19 pandemic in March 2020 due to high infectivity and rapid dissemination of the virus worldwide. COVID-19 belongs to the family of beta-coronaviruses [1]. SARS-CoV and MERS-CoV (Middle East Respiratory Syndrome Coronavirus) also belong to this family. These viruses are genetically identical due to single-stranded ribonucleic acid (RNA) in their genome. Like other coronaviruses, SARS-CoV-2 mainly affects the respiratory system and often affects the other systems [1].

The patients in COVID-19 generally present with fever, cough, and respiratory symptoms [2,3]. Involvement of the gastrointestinal system (GI) has also been reported. Diarrhea, abdominal pain, nausea, and vomiting are the predominant GI manifestations reported in infected COVID-19 patients [2,3]. Moreover, stool specimens and swabs from the anal region in the affected individuals also contained SARS-CoV-2 [4]. Interestingly, SARS-CoV-2 has also been identified in the feces of infected individuals, even after respiratory tract virus clearance. Furthermore, earlier studies reported that angiotensin-converting enzyme 2 (ACE2) receptors are responsible for viral adhesion and access to the host cell, and the GI tract has an abundance of ACE 2 receptors [5]. All above indicate that SARS-CoV-2 can infect and replicate within the GI tract, warrant the necessary treatment, management, and infection control. Hence, there is a dire need for rapidly growing data on this potentially fatal virus.

The respiratory manifestations are highlighted by infected patients and healthcare staff for testing and preventive isolation provisions, following the recommended guidelines and worldwide effort to halt the virus spread and alleviate its effects on the worldwide population [6]. The meta-analysis objective is to measure whether GI manifestations warrant the testing for SARS-CoV-2 alone or in combination with lung manifestations and the association of GI manifestations with severe disease and mortality. 

## Materials and methods

Search strategy and study design

**Figure 1:**
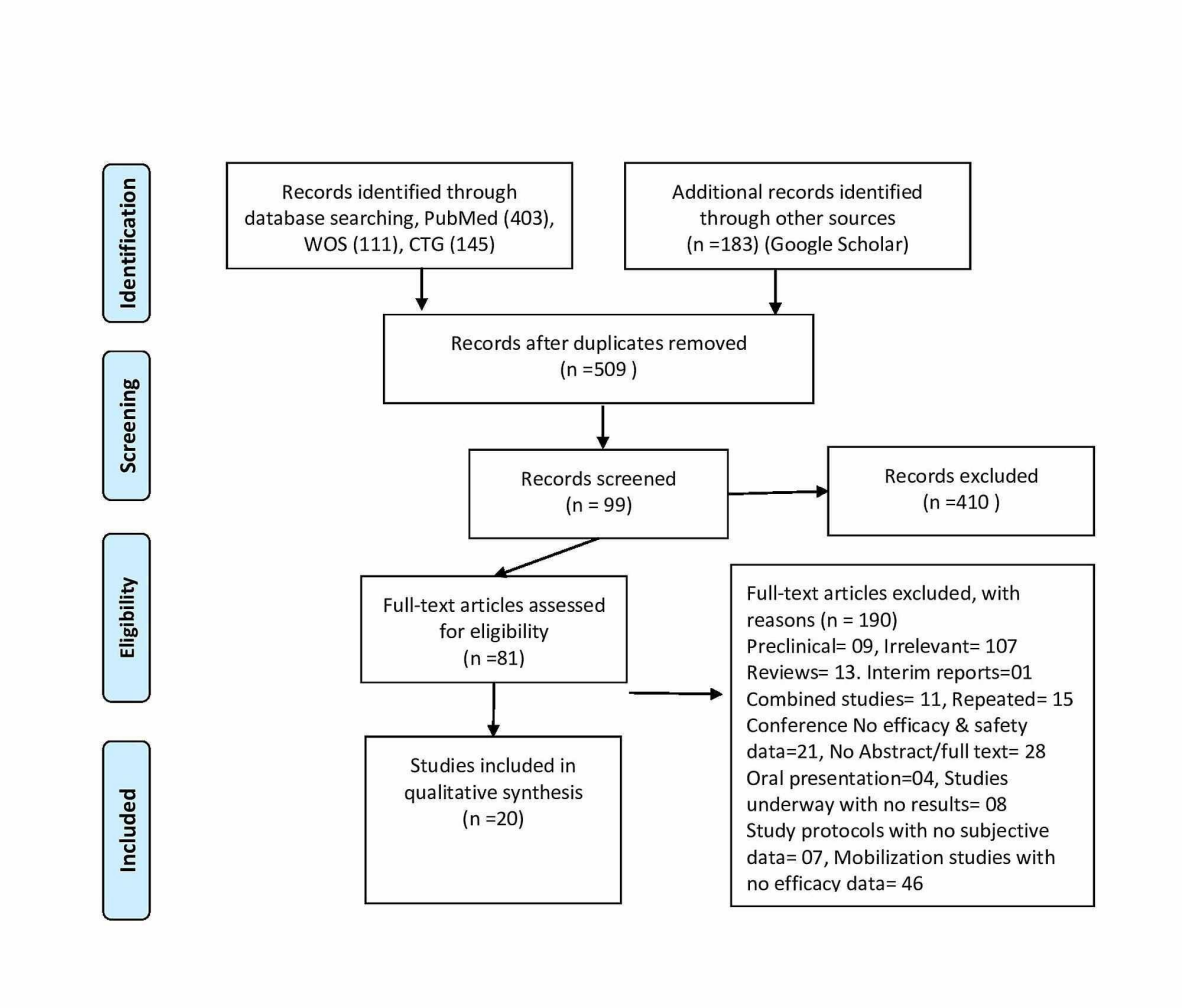
PRISMA flow chart PRISMA, Preferred Reporting Items for Systematic Review and Meta-Analyses; WOS, Web of Science; CTG, Clinicaltrials.gov.

We conducted a literature search using four databases (PubMed, Web of Science, Google Scholar, and Clinicaltrials.gov). Our search stratagem included MeSH (Medical Subject Headings) terms and keywords for COVID-19 and the GI system from the date of inception to October 2020. After excluding duplicates, review articles, and non-relevant articles, we included 20 studies out of 842 articles, reporting GI manifestations in COVID-19 patients as per PRISMA (Preferred Reporting Items for Systematic Review and Meta-Analyses) guidelines (Figure 1). We included any study reporting GI manifestations in a sample of more than five COVID-19 positive patients, including hepatic abnormalities with or without respiratory manifestations. Three authors independently reread each article for inclusion and exclusion criteria and pull out the data accordingly.

Study characteristics and selection of studies

All the articles were exported to Endnote version 8.0 (Clarivate, Philadelphia, USA). Our search identified 842 articles based on databases search, of which 20 met the inclusion criteria (Table 1). Most of the studies focused on adult patients in the hospital setting. Some studies included hospitalized subpopulations, such as medical staff, family clusters, pregnant, or critically ill patients. All defined COVID-19 cases were exclusively positive by polymerase chain reaction (PCR) taken through the upper respiratory swab. The language restrictions were not imposed. One author removed the duplicates. Two authors reviewed the reference list of screened articles manually, and two authors reviewed the included articles independently. The inclusion criteria involve observational studies with a retrospective design fixated to COVID-19 and GI manifestations. 

**Table 1:** Characteristics of included studies GI, gastrointestinal; COVID, coronavirus disease; LFTs, liver function tests; CT, computed tomography; ICU, intensive care unit; N/A, not applicable.

Author	Location	Study patient subset	COVID-19 patients	Age in median (years)	male (%)	Any GI symptom	Elevated LFTs	Severe COVID-19
Chen H [7]	China	N/A	21	56	81	4	6	11
Chen Q [8]	China	N/A	9	42.1	55.5	2	N/A	4
Guan WJ [9]	China	N/A	1099	47	58.1	42	158	173
Huang C [10]	China	Patients with COVID pneumonia	38	49	73	1	15	13
Hajifathalian K [11]	USA	NA	1059	61.1	57.7	350	657	NA
Jin X [1]	China	N/A	651	45	50.8	74	N/A	64
Li K [12]	China	Patients with respiratory symptoms who had a CT	83	45.5	53	7	N/A	25
Lian J [5]	China	N/A	465	45	52.26	36	99	49
Liu F [13]	China	N/A	10	42	40	3	N/A	5
Red [3]	USA	NA	318	63.4	54.7	195	NA	NA
Wan Y [14]	China	NA	230	47.9	56	49	N/A	61
Xia XY [15]	China	Familial cluster	10	56.5	60	2	N/A	3
Yang X [16]	China	ICU patients with COVID pneumonia	52	59.7	67	2	N/A	52
Zhang H [2]	China	N/A	505	51.2	45.1	164	N/A	92
Zhang J [6]	China	NA	663	55.6	48.4	61	171	409
Zhang JJ [2]	China	Patients with respiratory symptoms	140	57	50.7	55	N/A	58
Zhang R [17]	China	Patients with COVID-19 pneumonia	120	45.4	36	10	N/A	30
Zhao XY [18]	China	N/A	91	46	53.8	14	N/A	30
Zhou Z [19]	China	Patients with COVID-19 pneumonia	254	50	45.3	46	NA	NA
Pan L [20]	China	NA	204	52.9	52	101	0	1

Data extraction and quality of evidence assessment

The data was extracted from the included articles by using a standard excel sheet. Information on the first author, country of the study, publication year, mean age of the patients, gender ratio, the number of GI and hepatic manifestation, and the number of patients with severe COVID-19 were identified and extracted. Newcastle-Ottawa Quality Assessment Scale (NOS) was used to determine the quality of study design, analysis, and reporting results. The data was assessed across the four domains, such as study population selection, exposure, and outcome.

Statistical analysis

Our search included 20 articles out of 842 articles from different countries (Table 1). We estimated the pooled ratios of the patients who experienced GI manifestations for COVID-19 patients and the patients with hepatic manifestations. We measured the odds ratio (OR) of severe COVID-19 based on the presence of GI manifestation versus non-GI manifestations. We also calculated the OR of mortality in infected patients with GI and hepatic manifestations. Using the Cochrane RevMan version 5.4, a compute pooled analysis using the random effect model was performed. The heterogeneity was estimated by the I^2^ test. I^2^ statistics were categorized by the total variation in the percentage of effect size, which can be related to heterogeneity. Values greater than 50% and 70% were measured as moderate to high heterogeneity, respectively. 

## Results

**Figure 2:**
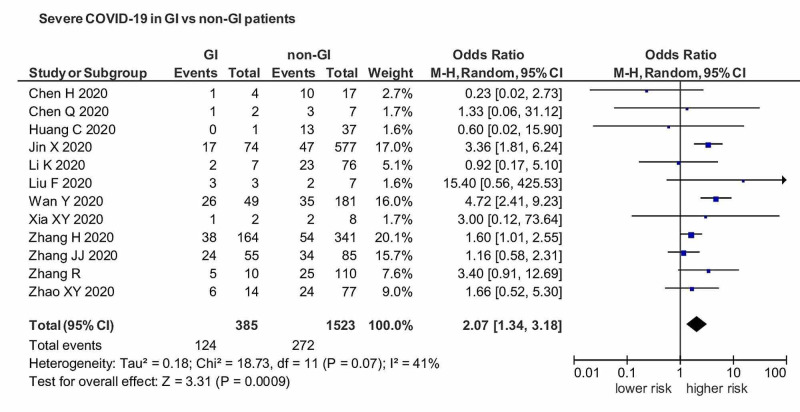
OR of severe COVID-19 in patients with GI manifestations versus non-GI manifestations. OR, Odds ratio; COVID, coronavirus disease; GI, gastrointestinal.

Our study included a total of 6,022 patients from different countries. Eighteen studies were included from China, and two studies were outside of China. The patients have a median age of 49.5 ± SD6.48, 53% were male, and 47% were female. Diarrhea, nausea, abdominal pain, and vomiting were included in the definition of GI manifestations. Anorexia was not included in the description of GI symptoms. A severe case of COVID-19 includes those patients that require hospitalization, fever more than 101^o^F, and symptomatic for the last ten days. A pooled analysis of severe COVID-19 in patients with GI manifestations versus non-GI manifestation showed an OR of 2.07 (95% CI: 1.34-3.18) with I^2^=41% (Figure 2). 

**Figure 3:**
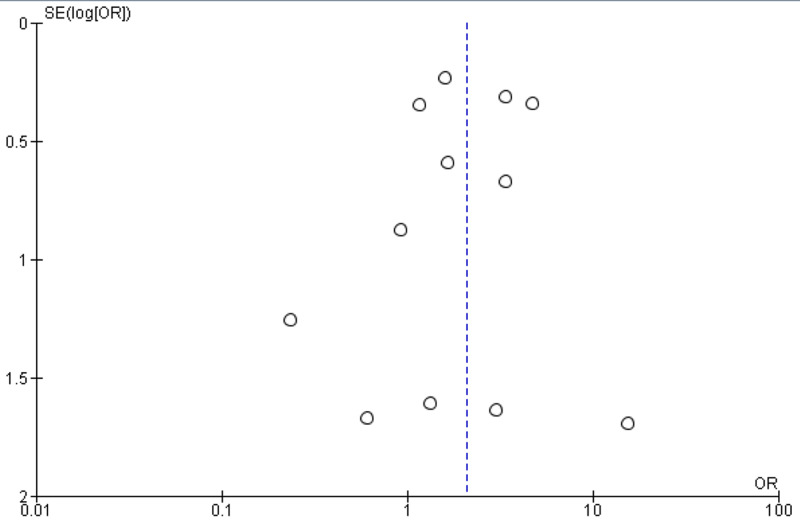
Funnel plot of comparison of severe COVID-19 in GI versus non-GI patients COVID, coronavirus disease; GI, gastrointestinal.

Results showed a significant association of GI manifestations with severe COIVD-19 (p<0.05). The funnel plot revealed a remarkable association between GI manifestation and severe COIVD-19 infection (Figure 3).

**Figure 4:**
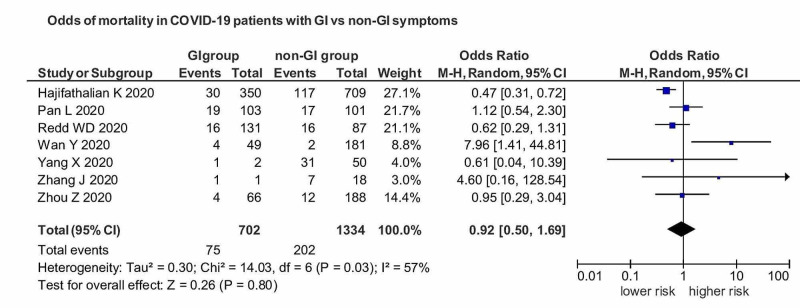
OR of mortality in COVID-19 patients with GI manifestations versus non-GI manifestations. OR, Odds ratio; COVID, coronavirus disease; GI, gastrointestinal.

There was a decreased risk of mortality in COVID-19 patients with GI manifestations versus non-GI manifestation with an OR of 0.92 (95% CI: 0.50-1.69) (I^2^=57%), but not of significant association (p=0.80) (Figure 4).

**Figure 5:**
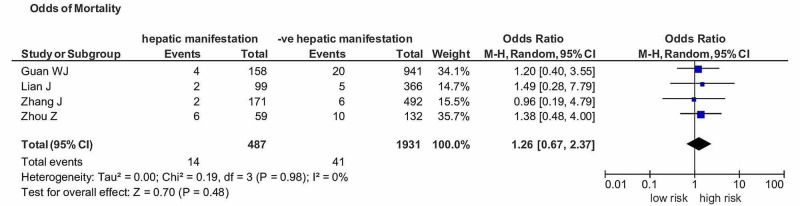
OR of mortality in COVID-19 patients with hepatic manifestations versus non-hepatic manifestations. OR, Odds ratio; COVID, coronavirus disease

We also estimated the pooled analysis of hepatic manifestations in COVID-19 patients. Any patient who had an abnormality in the liver study panel was included in the definition of hepatic manifestation. There was an increased risk of mortality in COVID-19 patients and hepatic manifestations with an OR of 1.26 (95% CI: 0.67-2.37) (I^2^=0%) but not of significant association (p=0.48) (Figure 5).

## Discussion

To the best of our knowledge, our study is one of the most extensive studies manifesting GI involvement in severe COVID-19, with more than 6,000 patients from 20 articles. COVID-19 is causing a major pandemic due to its high infectivity and constitutes a global health crisis. The patients are commonly present with respiratory manifestations. However, many patients also manifest nausea, vomiting, abdominal pain, and diarrhea. This analysis pooled the association of severe COVID-19 with GI manifestations and the risk of GI involvement in the death of COVID-19 patients. Pooled analysis via random effect model revealed an increased risk of severe COVID-19 in patients manifesting GI symptoms with an OR of 1.98 (95%CI: 1.25-3.14) (p=0.004) (I^2^ =47%). Odds of mortality in CVOID-19 with GI manifestation and hepatic abnormalities included 0.92 (95% CI: 0.50-1.69) (I^2^=57%) and 1.26 (95% CI: 0.67-2.37) (I^2^ = 0%), respectively. 

Cheung et al. revealed that 17.6% of the total 4243 infected patients had GI manifestation [21]. Another study reported that the prevalence of GI manifestation ranged from 2%-50% [22]. Even some patients with vomiting and severely ill patients had abdominal pain and GI bleeding [22]. The incidence of GI manifestations in patients with severe COVID-19 is conflicting. Fang et al. underlined that both stable and severe patients have high GI symptoms, and their prevalence was found to be 85% and 79%, respectively [23]. A similar inclination was noted in an extensive study of 1,099 patients, which underlined no differences in the percentage of GI manifestations in severe versus non-severe COVID-19 cases [9].

In contrast, a recent study reported a significantly higher prevalence of GI manifestations in critically ill and hospitalized patients [24]. Another analysis by Dorrel et al. reported a high prevalence of GI manifestations in severe COVID-19 patients [25]. A similar trend was noted in our study with more prevalence of GI manifestations in severe COVID-19 patients. However, none of the studies observed GI manifestations as a prognostic factor such as mortality. Our research found a decreased risk of mortality in patients with GI manifestations if we compare the positive patients with GI manifestations and those without GI manifestations. However, the results were not significant. The opposite but non-significant results were observed in cases of hepatic manifestations with COVID-19.

In MERS-CoV disease, the patients also manifested GI symptoms ranging from 11.5-32%. During the endemic of SARS-CoV disease, nearly 20% of the infected patients were reported to have GI manifestations [26,27]. Remarkably, recent studies stated that coronaviruses show tropism to the gastrointestinal tract, which might elaborate the frequent GI involvement in coronavirus diseases. The detection of viral RNA in stool specimens of infected individuals has been underlined in the literature. The electron microscope on biopsy or autopsy samples of the gut revealed active replication in affected patients [26]. Similarly, GI involvement in MERS-CoV has also been reported as the human epithelial cells of the GI tract is highly susceptible to MERS-CoV [2]. Since the genome sequence of SARS-CoV-2 has approximately 80% identity with SARS-CoV, gut involvement by SARS-CoV-2 is not astounding. Our study reported a higher prevalence of GI manifestations in patients with severe COVID-19 than those with the less critical disease. Our study's finding might have possible prognostic inferences, justifying the close monitoring of infected patients with GI involvement. 

Apart from the respiratory symptoms, GI and hepatic manifestations in COVID-19 could be elucidated by ACE2 cell receptors. The previous studies have justified the mechanism of GI involvement through binding of viral glycoproteins, spike (S) protein to cellular ACE2 receptors of the host cell, responsible for viral entry into the host cell [5]. Besides this, it has also been observed that the receptor-binding domain on COVID-19 has a high affinity to human ACE2 receptors [4]. ACE2 receptors are highly expressed in alveolar lung cells, GI epithelial cells, especially in the small and large intestine, neuronal-glial cells, and epithelial cells of the kidney [27]. This data gives valuable insight into the receptor-mediated entry of COVID-19 into the gut cells and provides a strong base for its potential transmission through the fecal matter. GI involvement in COVID-19 may be responsible for enhanced exposure and significant viral load; thus, GI involvement may be a proxy for more critically ill patients.

Our study has a direct impact on viral infectivity. Towards this notion, Zhou et al. stated that SARS-CoV-2 could remain viable in aerosols for hours and could be stable for at least 72 hours on stainless steel and plastic surfaces [28]. Viral excretion in fecal matter, its environmental steadiness would result in the rapid spread of COVID-19 from human to human, as stated during the endemic of SARS-CoV in Hong Kong [29]. GI involvement in COVID-19 and other systems involvement may warrant the organized hospital policies, such as the use of anal swab for COVID-19 testing before discharging the patient. Undoubtedly, our study results are significant and should be taken seriously into account in our fight against COVID-19.

Our meta-analysis has remarkable limitations. As an analysis of many studies, there is significant heterogeneity, which is reduced in subgroup analysis. One of the limitations is generalizability, as most of the included studies are from China and conducted in hospitalized settings. No reviews had a low bias risk. This is due to the absence of COVID-19 hostile comparison groups, suboptimal depth of GI manifestations, and lack of proper follow-up of the infected patients. Recently, flawed methodology and systematic approach have been reported in many studies, driven by incredulous healthcare organizations needing rapid data dissemination with high-quality peer review [30]. A significant association of severe COVID-19 with GI manifestations in our study will greatly impact GI practice.

## Conclusions

We perceive that COVID-19 has a significant association with GI manifestations. An extraordinary catalog of suspicion for such patients will be pertinent to prevent or, at least, minimize the contact to high-risk patients. The above analysis of the gastrointestinal manifestations of COVID-19 will help the gastroenterologist to have a crucial preparation, which is of supreme importance to prevent infections. The significant role of digestive manifestations in COVID‐19 is precise, but many knowledge gaps regarding their pathophysiology, management, and predictive value persist. Associated GI manifestations in severe COVID-19 have implications for both patient care and infection control. Our study highlights the need for high-quality data from recent literature, including the patients from community settings, and further explores the underlying mortality causes.
